# Defined culture conditions improve functional properties of mature iPSC-derived macrophages for therapeutic screening

**DOI:** 10.1186/s13287-026-05009-1

**Published:** 2026-04-11

**Authors:** Maximilian Schinke, Ingrid Gensch, Tina Rietschel, Ariane Hai Ha Nguyen, Hortense Slevogt, Lothar Jänsch, Nico Lachmann

**Affiliations:** 1 Fraunhofer Institute for Toxicology and Experimental Medicine ITEM, Nikolai-Fuchs-Straße 1, 30625 Hannover, Germany; 2 Helmholtz Centre for Infection Research, Inhoffenstraße 7, 38124 Braunschweig, Germany; 3 Department for Pediatric Pneumology, Allergology and Neonatology, Hannover Medical School, Carl-Neuberg-Str. 1, 30625 Hannover, Germany; 4 Department of Respiratory Medicine and Infectious Diseases, Hannover Medical School, Carl-Neuberg Str. 1, 30625 Hannover, Germany

**Keywords:** hiPSC, Macrophages, Drug screening, Cell manufacturing, Physiologic media, RNA-Seq, Surfaceome, cell surface proteomics

## Abstract

**Background:**

Generation and use of human macrophages in vitro are essential for a variety of (therapeutic) applications. Recently, there has been growing interest in macrophages derived from induced pluripotent stem cells (iPSC-Mac), which has led to the development of numerous differentiation protocols. These protocols typically involve a stepwise differentiation process using well-defined culture media, though the final differentiation stage often relies on undefined animal serum components, such as fetal bovine serum (FBS). This study aimed to elucidate protocol-dependent effects on iPSC-Mac usability.

**Methods:**

We assessed the impact of a serum-supplemented medium (RPMI + FBS) compared to a fully defined medium (X-VIVO 15™) on the function of terminally differentiated human iPSC-Mac. Upon harvest, the cells were differentiated for three to four days in the respective media. Subsequently, phenotypic analysis was performed by microscopy and flow cytometry, as well as transcriptome and proteome analysis. Functional responsiveness to stimuli such as polarizing cytokines and lipopolysaccharide (LPS) was evaluated using flow cytometry and ELISA assay. The pharmaceutical screening potential was examined using secondary stimuli with dexamethasone.

**Results:**

While both media compositions effectively produced fully functional human macrophages, we observed significant differences in their stage of activation and their functional responsiveness. Macrophages differentiated under the defined, serum-free conditions showed a more neutral activation state, an enhanced cholesterol metabolism, and were more sensitive to post pro- or anti-inflammatory stimulation compared to serum-grown cells. Most important, iPSC-Mac from these cultures also exhibit a more robust and reproducible response to co-stimulatory signals with IFNγ or LPS in combination with the immune-suppressive agent dexamethasone. In contrast, serum-grown iPSC-Mac demonstrated a more pre-activated state, with noteworthy background levels of interleukin 6 (IL-6). Upon stimulation, these cells showed reduced sensitivity and responsiveness towards secondary signals, underscoring potential challenges when using FBS-based media for drug screening or immunomodulatory assessments.

**Conclusions:**

The enhanced responsiveness to co-stimulatory signals makes iPSC-Mac from defined cultures more suitable for testing immunomodulatory drugs, novel bio-assays and in vivo applications.

**Supplementary Information:**

The online version contains supplementary material available at 10.1186/s13287-026-05009-1.

## Introduction

During the last decade, human macrophages have increasingly become the subject of many research topics. Through their appearance as tissue-resident macrophages (TRM) within the whole human body [1], macrophages are getting more and more attention regarding their involvement in different disease entities like fibrosis, cancer, inflammatory diseases and infection [2–4]. Macrophages are a key cell type that acts as an important pro- and anti-inflammatory mediator balancing immune responses and simultaneously contributing to organ homeostasis [5].

Given their critical role in various diseases, pharmacological modulation of macrophage activity represents a promising therapeutic strategy. In vitro screening and validation of novel therapeutic options has been thoroughly implemented at varying degrees of complexity. These attempts comprise the direct targeting to alleviate hyperinflammatory conditions [6, 7] or monogenetic disorders [8, 9]. Additionally, simple co-culture systems have been implemented for the evaluation of therapeutic approaches targeting cancer or fibrosis [10, 11]. Conversely, more complex model systems are progressing, involving multiple cell types that are used to generate immunocompetent organoid structures to better mimic in vivo conditions [12, 13].

Despite their involvement in different malignancies, macrophages hold another important characteristic. Through the expression of different Toll-like receptors (TLR) or pattern recognition receptors (PRR), macrophages serve as sensory cells and have become a versatile tool in toxicological studies [14, 15]. The assessment of chemicals or materials for toxicity, as well as the screening for endotoxic contamination relies on the activation of monocyte/macrophage populations, resulting in the secretion of pro-inflammatory cytokines like interleukin 6 (IL-6), tumor necrosis factor alpha (TNFα) or IL-1β, which can be measured to assess the hazardous potential [16].

The growing interest in human macrophages has thus led to a greater demand of consistent cell sources. One particularly promising approach to this challenge are induced pluripotent stem cells (iPSCs). iPSCs have successfully been used to generate different kinds of somatic cells, including macrophages. Due to their primitive transcriptional profile, addition of further stimulants or the co-cultivation with other cell types, facilitates their differentiation towards TRM-like cells [17, 18] and have intensively been studied as a screening tool for pharmacological investigations [19]. For the generation of iPSC-Mac, different protocols have been described. They are mostly founded on an embryoid body (EB) -based approach that generates myeloid cells following hematopoietic specification [20, 21]. The protocols used to date rely on serum-free media to expand iPSCs and generate hematopoietic organoids. After harvesting the iPSC-Mac, the protocols follow different concepts. They either continue to use defined media such as X-VIVO™ 15 or OXM [19, 22–25], utilize serum replacement strategies such as knockout (KO) serum [26] or human platelet lysate medium (HPLM) [27], or follow cultivation protocols known from primary macrophages based on Roswell Park Memorial Institute Medium (RPMI) or Dulbeccos Modified Eagle Medium (DMEM) medium supplemented with fetal bovine serum (FBS) [20, 28–34]. Given the utilization of macrophages for different immunological questions, medium composition strongly influences the behavior of the cells and thus the result of any read-out. Antonson et al. highlighted tremendous effects of DMEM on PBMC-derived macrophages due to its lack of non-essential amino acids. In the context of iPSC-Mac, Bussi et al. described the usage of HPLM as a more physiological serum-replacement, which led to a decreased lipid metabolism, an increase in metabolic polarization, and lysosomal and mitochondrial activity [27]. These changes significantly altered replication of *Mycobacterium tuberculosis* after infection, highlighting the importance of medium composition for understanding host-pathogen interaction. In this line, the usage of FBS is particularly noteworthy. Small molecules in FBS can induce the secretion of proinflammatory cytokines in epithelial cells [35, 36]. Along with the significant variability between different LOTs [36], the usage of animal serum-containing medium requires careful consideration when used for evaluating or assessing the immunomodulatory/immunogenic potential of compounds on macrophages. Moreover, ethical concerns regarding animal welfare arise when considering the reliance on animal-derived products in scientific research. Nevertheless, various recent publications are still using animal serum-containing media for the final differentiation and utilization of iPSC-Mac [20, 28–34]. Therefore, the following work highlights the manufacturing of iPSC-Mac for drug- and toxicological screening purposes under serum-free and serum-containing conditions and illustrates the consequences of both cultivation strategies on cell phenotype, function and responsiveness.

## Materials and methods

### Human iPSC cultivation

Experiments were performed utilizing two different already established iPSC lines from healthy donors: iPSC line#1 (female, hCD34iPSC11, MHHi015-B (RRID: CVCL_ZX24)) and iPSC line#2 (female, SCTi003-A (RRID: CVCL_C1W7)). Both iPSC lines were derived from peripheral blood, reprogrammed using a lentiviral or Sendai vector, respectively. The donor patients provided written informed consent to participate in this study. Further information about the donors and ethics can be found online in the Human Pluripotent Stem Cell Registry (https://hpscreg.eu/).

The iPSC lines were expanded in feeder-free monolayer culture on Geltrex™-coated tissue culture plates (diluted 1:400 in DMEM/F-12; 1 ml sufficient for 5 cm^2^, (both Gibco)) and cultured under standard humidified conditions at 37 °C and 5% CO_2_ as previously described [9]. The passaging step involved a pre-wash with Dulbecco’s Balanced Salt Solution (D-PBS) without Ca^2+^/Mg^2+^ (Gibco) and detachment with Accutase^®^ (Sigma-Aldrich) for 3–5 min at 37 °C. Dulbecco’s Modified Eagle Medium/Nutrient Mixture F-12 (DMEM/F-12) was added to dilute the Accutase^®^ and the cell suspension was transferred into a conical tube for subsequent centrifugation at 100 xg for 5 min, followed by resuspension of the cell pellet and seeding in Essential 8 (E8)-medium (Essential 6 (E6) medium (DMEM/F-12, 64 mg/L ascorbic acid 2-phosphate, 14 µg/L sodium selenite, 543 mg/L NaHCO_3_, 20 mg/L insulin, and 10.7 mg/L human recombinant transferrin (all from Sigma-Aldrich)), freshly supplemented with 100 ng/mL human basic fibroblast growth factor (hbFGF) and 2 ng/mL human transforming growth factor beta 1 (hTGFb1) (both from PeproTech)), supplemented with 10 µM Y-27632 dihydrochloride (ROCK-Inhibitor (RI); Tocris). The medium was exchanged to RI-free E8-medium after 48 h (day 2) and cells were passaged again after 72 h (day 3) for a maximum of 6 passages.

### Hematopoietic differentiation of iPSC to macrophages

To initiate inoculation of myeloid cell forming complexes (MCFC), 5 × 10^5^ iPSCs were seeded in 3 mL of mesoderm priming Ia medium (E6-medium supplemented with 50ng/mL hbFGF, 1 ng/mL hTGFb1, 50 ng/mL human vascular endothelial growth factor (hVEGF) and human bone morphogenetic protein 4 (hBMP4), 20 ng/mL human stem cell factor (hSCF) (all from PeproTech)) and 10 µM RI per well of a CELLSTAR^®^-6-well plate (Greiner Bio-one) and placed on an orbital shaker (OMNI Life Science) at 70 rpm. On day 2, the medium was changed carefully to mesoderm priming Ib medium (E6-medium supplemented with 50 ng/mL hVEGF and hBMP4, 20 ng/mL hSCF and 10 µM RI) while increasing the shaking speed to 85 rpm. On day 4 after inoculation, supernatant was removed and 3 mL of mesoderm priming II medium (E6 medium supplemented with 50 ng/mL hVEGF and hBMP4, 20 ng/mL hSCF and 25 ng/mL human interleukin 3 (hIL-3; Peprotech)) was added and refreshed at day 7. Hematopoietic differentiation was started at day 10 by transferring the formed MCFC of one well to 3–4 wells of a new 6-well tissue culture plate and changing the old medium of each well to 2 mL of differentiation medium (X-VIVO™ 15 (Lonza), 100 U/mL penicillin-streptomycin, 2 mM L-glutamine, 50 µM β-mercaptoethanol (all from Gibco) supplemented with 25 ng/mL hIL-3 and 50 ng/mL human macrophage colony stimulating factor (hM-CSF, Peprotech)). In the first week, medium was changed twice. Continuous macrophage (iPSC-Mac) production starts from d7 onwards, and cells can be harvested every 7 days during a medium change, by filtering through a 70 μm gaze (Sarstedt) before further analysis/experiments.

### Terminal differentiation of iPSC-Mac

For final maturation, iPSC-Mac were collected in a conical tube and centrifuged at 300 xg for 5 min before seeding into either X-VIVO medium (X-VIVO™ 15, 100 U/mL penicillin-streptomycin, 2 mM L-glutamine, 50 µM β-mercaptoethanol supplemented with 50 ng/mL hM-CSF), or RPMI medium ((RPMI + FBS), RPMI 1640 medium + L-glutamine (Gibco), 10% heat-inactivated fetal bovine serum ((FBS Superior, lot no. 0001647073); Sigma Aldrich) and 100 U/mL penicillin-streptomycin supplemented with 50 ng/mL hM-CSF), or RPMI medium without FBS ((RPMI-FBS); RPMI 1640 medium + L-glutamine and 100 U/mL penicillin-streptomycin supplemented with 50 ng/mL hM-CSF) respectively and cultured for 3 days under standard humidified conditions at 37 °C and 5% CO_2_.

### Measurement of glucose and lactate concentrations

The concentration of glucose and lactate in the supernatant of the different terminally differentiated iPSC-Mac was determined using the Biosen C-Line device (EKF Diagnostics) following the manufacturer’s instructions with 20 µL sample volume.

### Microscopic imaging und cytospin stainings

Terminally differentiated iPSC-Mac of the different conditions were observed under the Evident CKX53 microscope and imaged at 4x, 10x, 20x and 40x magnification. Cell counts were performed using a Neubauer chamber and normalized to the initial number of seeded cells.

To apply cells to a slide for staining, 2 × 10^4^ terminally differentiated iPSC-Mac were collected and resuspended in 120 µL PBS and centrifuged at 700 rpm for 10 min using a Cellspin I Cytocentrifuge (THARMAC). The slides were air-dried and then stained with 0.25% (w/v) of May-Grünwald solution (Carl Roth) for 5 min and washed three times with distilled water. The second staining round was performed using 5% GIEMSA solution (Carl Roth) for 20 min. Next, the slides were thoroughly washed three times with distilled water. Stained and air-dried cells were fixed with ROTI^®^ Histokitt mounting solution (Carl Roth) and imaged at the Evident CKX53 microscope.

### Phagocytosis assay using pHrodo™ BioParticles™

To evaluate the phagocytic activity of freshly harvested iPSC-Mac, 2.5 × 10^5^ cells per well were seeded into a 12-well tissue culture plate in 500 µL X-VIVO medium and incubated with 10 µL of pHrodo™ Red E. coli BioParticles™ (ThermoFisher Scientific). A sample without particles served as a control. After 2 h, the cells were collected, washed with PBS and the phagocytic events were quantified using flow cytometry (CytoflexS).

### Measurement of reactive oxygen species (ROS assay)

To evaluate the production of ROS, the iPSC-Mac were divided into three conditions with each 3 × 10^5^ cells in 500 µL Hanks’ Balanced Salt Solution (HBSS) solution with Ca^2+^/Mg^2+^ (Gibco): 1: unstained; 2: stained with Dihydrorhodamine 123 ((DHR); Sigma-Aldrich); 3: stained with DHR and stimulated with the ROS inducer phorbol myristate acetate ((PMA); Sigma-Aldrich). To prepare the cells for the staining and/or the stimulation, the resuspended cells were incubated at 37 °C for 5 min at 400 rpm on a shaker. Afterwards, PMA (400 ng/mL final concentration) was added to condition 3 for stimulation and incubated at 37 °C for 5 min at 400 rpm. Next, DHR (500 ng/ml final concentration) was added to the conditions 2 and 3 to stain the cells and incubated at 37 °C for 15 min at 400 rpm. Lastly, the samples were washed with PBS and evaluated for their ROS production by measuring the fluorescent signal of the oxidized rhodamine using flow cytometry (CytoflexS).

### Polarization and stimulation of terminally differentiated macrophages

Since macrophages react to different external stimuli by polarization towards a certain activation state, 2.5 × 10^5^ iPSC-Mac per well were seeded in a 24-well tissue culture plate under the different conditions of terminal differentiation. On day 3, the cells were stimulated with 25 ng/mL human interferon-gamma (IFNy) to induce pro-inflammatory M1 polarization, 10 ng/mL human interleukin-4 (IL-4) for anti-inflammatory M2a polarization or 10 ng/mL hTGFb1 and human interleukin-10 (IL-10) for anti-inflammatory M2c polarization (all from Peprotech) in 250 µL of the respective medium for 24 h. Non-stimulated cells served as a control. After 24 h, cells were harvested and analyzed by flow cytometry (CytoflexS).

### Flow cytometry of iPSC-Mac surface marker expression

Surface marker expression of freshly harvested or terminal differentiated and stimulated iPSC-Mac was assessed using flow cytometry. Therefore, cells were collected and washed with PBS. To block nonspecific antibody binding and perform a life-dead staining, cells were incubated with FcR Blocking Reagent (1:50 in PBS; Miltenyi Biotec #130059-901) and Zombie Aqua™ Fixable Viability Kit (1:250 in PBS; Biolegend #423101) for 10 min at room temperature. Thereafter, the cells were stained with fluorescently labelled antibodies diluted in FACS buffer (PBS, 2 mM ethylenediaminetetraacetic acid ((EDTA); Carl Roth) and 2% FBS) for 20 min at 4 °C in the dark. Detailed information on the following anti-human antibodies and dilutions can be found in the supplementary table S1:

CD45-BV421 (Invitrogen #404-0459-42), CD14-PE (Invitrogen #12-0149-42), CD11b-PE-Cy7 (Biolegend #301322), CD163-APC (Biolegend #333610), SSEA4-FITC (Biolegend #330410), CD206-BV421 (Biolegend #321126), CD64-FITC (Biolegend #305006), CD86-PE (Invitrogen #12-0869-42), HLA-DR-APC (Biolegend #307610), CD66b-APC (Invitrogen #17-0666-42), CD16-PE-Cy7 (Invitrogen #25-0168-42).

All flow cytometry experiments were performed using a CytoFLEX S (Beckman Coulter). A minimum of 1 × 10^5^ events per staining were acquired and data were analyzed with FlowJo software v10.10.0 (BD Bioscience).

### Evaluation of iPSC-Mac sensitivity to anti-inflammatory treatment

In order to assess the sensitivity of iPSC-Mac towards anti-inflammatory treatment upon pro-inflammatory stimulus, 5 × 10^5^ cells per well were seeded into a 12-well tissue culture plate for 3 days under the different conditions of terminal differentiation. Subsequently, cells were stimulated simultaneously with 100 ng/mL lipopolysaccharide (LPS) and 1 µg/mL dexamethasone (both from Sigma-Aldrich) for 4 h. Then, the supernatants were collected and frozen at -80 °C for further quantification of human IL-6 secretion using an ELISA assay. A non-stimulated and the individually stimulated conditions served as controls. The concentration of secreted IL-6 was determined with the DuoSet ELISA kit (DY206, R&D Systems) according to the manufacturer’s instructions.

### Dose-escalation experiment of terminally differentiated iPSC-Mac with different concentrations of lipopolysaccharide

To determine the sensitivity of the various iPSC-Mac to the endotoxin LPS, 2.5 × 10^4^ cells per well were seeded in a 96-well tissue culture plate and terminally differentiated with X-VIVO medium, X-VIVO medium supplemented with 100 ng/mL human lipopolysaccharide-binding protein ((LBP); Acro biosystems) or RPMI + FBS medium for 3 days. The WHO 3rd international endotoxin standard ((WHO-LPS); NIBSC #10/178) was prepared in a serial dilution (200–3.125 pg/mL) in sterile water for injection (B. Braun Melsungen AG). On the day of stimulation, the old medium was completely removed from the wells and replaced with 20 µL of the respective WHO-LPS concentration and 240 µL of the respective culture medium. After 24 h incubation, the supernatants were collected and stored at -80 °C for further secretion analysis using the IL-6 DuoSet ELISA kit.

### RNA isolation and sequencing

For transcriptome analysis, 5 × 10^5^ hiPSC-Mac of iPSC line #2 per well of a 12-well tissue culture plate were terminally differentiated as previously described. Samples were prepared as two biological replicates. On day 3, the cells were washed with PBS and resuspended in RNA lysis buffer. RNA isolation was performed with the Direct-zol RNA Microprep Kit (Zymo Research) according to the manufacturer’s instructions. Quality control, mRNA sequencing library preparation (poly A enrichment), paired-end sequencing on a NovaSeq X Plus Series with a read length of 150 base pairs, raw data processing, and annotation were performed at Novogene GmbH (Munich, Germany). Data normalization and differential gene expression analysis was performed in R using *DESeq2* (version 1.44.0). Comparative analysis was conducted on genes with adjusted p-values < 0.05 and a log_2_ fold change (|log2FC|) > 0.9. Data were visualized using the *gplots* (version 3.2.0) and *ggplot2* (version 3.5.1) packages. Gene set enrichment analysis was performed using the R package *clusterProfiler* (version 4.12.6) with an adjusted p-value cut-off of 0.05. Additionally, enrichment was performed and visualized using the GSEA software [37, 38].

### Enrichment of glycoproteins for proteomic analysis

As described in the previous sections, 1 × 10^6^ iPSC-Mac were terminally differentiated in a 6-well tissue culture plate. After three days, the different cultivation conditions were washed several times with PBS and lysed in 2% SDS/PBS. Lysates were boiled for 5–10 min and stored at -80 °C until further use. Glycoprotein enrichment was performed as described previously [39]. Briefly, equal amounts of protein dissolved in 100 µL 2% SDS/PBS were centrifuged for 20 min at 20,000 xg. The supernatant was subjected to Pierce™ polyacrylamide spin-desalting columns (Thermo Fisher Scientific) equilibrated with 150 mM sodium acetate (pH 5.5) for buffer exchange. Carbohydrates were oxidized with 10 mM sodium periodate (Sigma-Aldrich) and incubated 30 min in the dark. Subsequently, another buffer exchange was performed with polyacrylamide spin-desalting columns equilibrated with PBS. The oxidized glycoproteins incubated overnight with Ultralink™ hydrazide resin beads (Thermo Fisher Scientific) in the presence of aniline as catalyst. The beads were washed five times with 0.5% SDS/PBS and one time with 8 M Urea/100 mM Tris-HCl pH 7.8. For the reduction and alkylation step, proteins were incubated for one hour at 37 °C with 50 mM dithiothreitol (DTT) and 30 min with 50 mM iodoacetamide in the dark. To remove non-glycoproteins from covalently linked glycoproteins, the beads were stringently washed: 3 × 8 M urea/100 mM Tris-HCl pH 7.8, 1 × 100 mM Tris-HCl (pH 7.8), 3 × 3 M sodium chloride (NaCl) in 100 mM Tris-HCl (pH 7.8), 3 × 100 mM sodium acetate 150 mM/NaCl (pH 3.0), 2x H_2_O, 3 × 30% acetonitrile, 2 × 50 mM ammonium hydrogencarbonate (ABC). After the final wash, the beads were resuspended in 20 µL ABC and digested with 1 µg trypsin overnight at 37 °C. The peptide-containing eluate was collected. The beads were eluted again with 50 mM ABC and 30% acetonitrile. All eluate collections were combined and dried. The beads were washed again as described above and digested with 1U PNGase F (Promega) at 37 °C overnight. The eluate containing deglycosylated N-linked glycopeptides was collected and dried as a separate fraction.

### Mass spectrometric analysis of the glycoproteome and processing of LC-MS data

Samples were dissolved in 0.1% formic acid. Next, 250 ng of the tryptic fraction and 15 ng of the PNGase Fraction were injected to a Dionex Ultimate 3000 HPLC system coupled to an Orbitrap Fusion™ mass spectrometer (Thermo Fisher Scientific). The general set-up was: spray voltage of 1.8 kV, heated capillary temperature of 300 °C, S-lens RF level of 60%, ion selection threshold of 50,000 counts for HCD. All peptides were loaded to a 200 cm C18 µPac™ analytical HPLC column (PharmaFluidics). Tryptic fractions were separated over a 160 min non-linear gradient (3%-90% acetonitrile) with a flow rate of 300 nL/min. Overview spectra were recorded with a cycle time of 1 s and a maximum injection time of 50 ms. The scan window was set to 400–1600 m/z with a resolution of 120,000. Selected precursors were fragmented with a higher energy collisional dissociation (HCD) of 30%. Ions were collected for MS2 spectra with a maximum injection time of 300 ms and AGC 2.5 × 10^4^. The PNGase fraction was analyzed with the following changes: Peptides were eluted from the analytical columns in an 80 min non-linear gradient. Selected precursors for MS2 spectra were collected with a maximum injection time of 500 ms and an AGC of 1.2 × 10^4^. Raw files were analyzed using the MaxQuant software (version 2.4.2.0) with default settings if not stated otherwise. For the protein identification and label-free quantification (LFQ), carbamidomethylation on cysteines was considered as fixed and methionine oxidation as variable modification. Additionally, asparagine deamidation was enabled for PNGase fractions. All peptides were used for protein quantification. Bioinformatic analysis was performed with Perseus software version 2.1.1.0. For this, identified and LFQ-quantified proteins were filtered to remove potential contaminants, reversed proteins and proteins only identified by site. Only proteins stably identified in both replicates of any condition were kept for further analysis. Missing values were imputed by normal distribution with a width of 0.3 and a down shift of 1.8. Gene ontology (GO) annotations for human proteins were added and pairwise comparisons of the different culture media were performed. Proteins were considered as significantly altered if the p-value was below 0.05.

### Statistical analysis

Statistical analysis and data visualization was performed using GraphPad Prism version 10. The type of t-test and analysis of variance (ANOVA) as well as post-hoc testing is indicated in the respective figure description. All experiments depict biological replicates from three or more different harvests as mean ± SD. Individual data points are plotted in addition, when they provided further value. Asterisks denote: ns – not significant; * – p-value < 0.05; ** – *p* < 0.01; *** – *p* < 0.001; **** – *p* < 0.0001.

## Results

### Human iPSC-Mac share morphological and phenotypic characteristics following terminal differentiation in different protocols

**Fig. 1:**
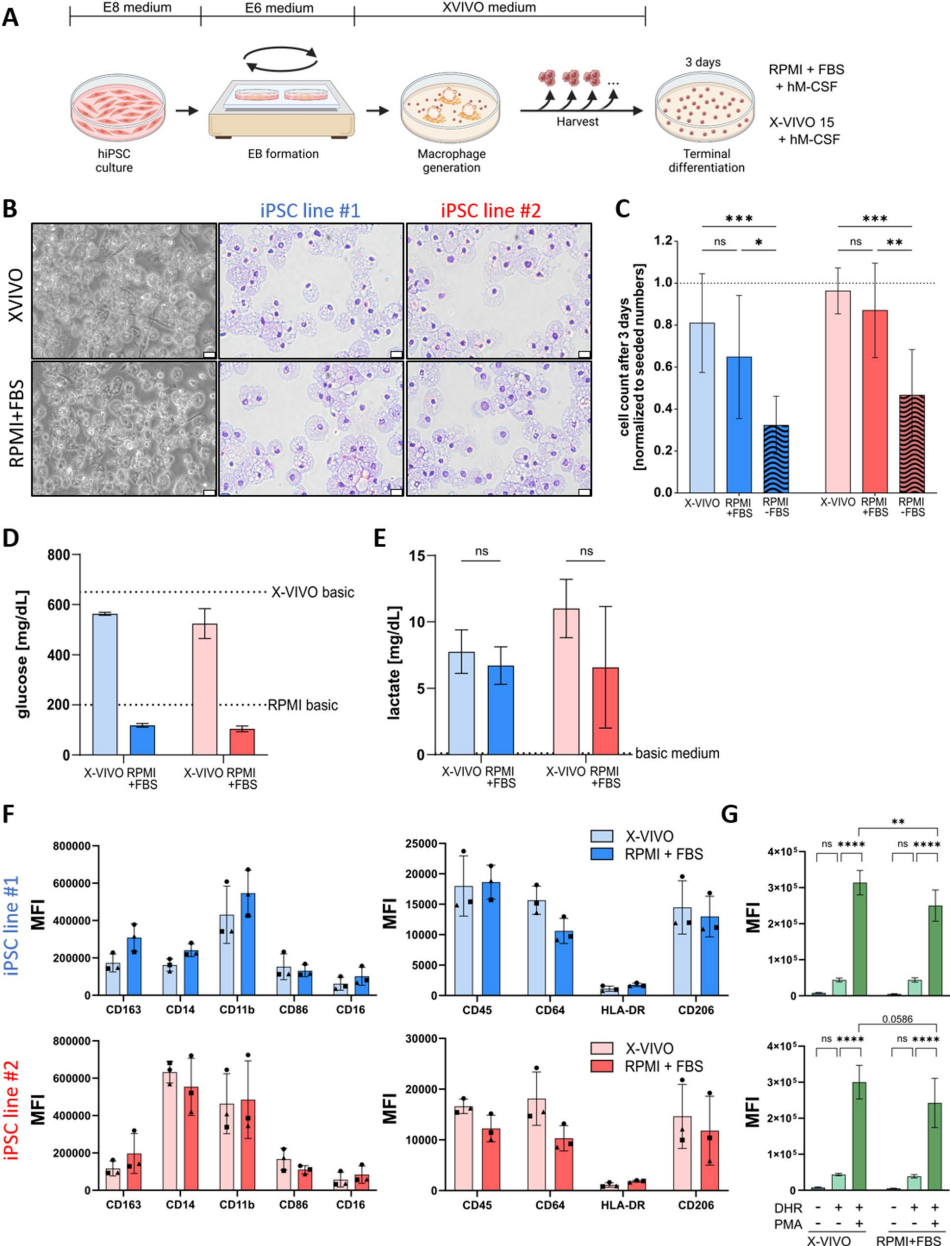
Phenotypic and morphological characterization of iPSC-Mac after terminal differentiation in different media. **A** Schematic overview of the generation of macrophages (iPSC-Mac) from human induced pluripotent stem cells (iPSC) and media used during terminal differentiation. **B** Representative microscope picture and cytospin stainings for the two different iPSC-Mac lines after three days of terminal differentiation in either X-VIVO + M-CSF or RPMI + FBS + M-CSF. **C** Number of viable cells counted after 3 days of terminal differentiation in respective media, normalized to the initial number of seeded cells. An additional condition includes RPMI without FBS (RPMI-FBS). Graphs show mean ± SD. Statistical analysis was performed using two-way ANOVA with Tukey’s multiple comparison post-hoc test, *n* = 7. **D** Remaining glucose concentrations and **E** lactate production after 3 days of terminal differentiation. Dotted lines indicate basal glucose and lactate concentrations. Unpaired t-test, both *n* = 3. **F** Histograms showing the overall and individual median fluorescence intensities (MFI) of different macrophage markers from iPSC line#1 (blue) and line #2 (red) upon 3 days of terminal differentiation in either X-VIVO or RPMI + FBS measured by flow cytometry. Markers with high MFI values are depicted on the left and markers with lower MFI separately on the right (*n* = 3). Representative flow cytometry histograms can be found in Supplementary Figure S3. **G** Production of reactive oxygen species (ROS) by macrophages on day 3 of differentiation. Bar graphs showing shifts in the MFI upon oxidation of Dihydrorhodamine (DHR) into its fluorescently active form by intracellular reactive oxygen species (ROS) following stimulation with phorbol myristate acetate (PMA). Graphs show mean ± SD. Statistical analysis was performed using two-way ANOVA with Tukey’s multiple comparison post-hoc test, *n* = 3

This study aims to manufacture iPSC-Mac for immunopharmacological or toxicological applications using two different, well-established protocols. We therefore differentiated two iPSC lines (iPSC line #1 and iPSC line #2, see method description) according to a standardized, previously published protocol under defined, serum-free and feeder-free conditions [9]. According to this protocol, hematopoietic organoids (hemanoids) were cultivated in tissue culture plates in X-VIVO™ 15 (X-VIVO) medium supplemented with M-CSF and IL-3. Although various alternative media formulations have been described recently [24, 27], differentiation media based on X-VIVO are still the most commonly used in serum-free protocols and have therefore been used in the present study [19, 20, 22, 23, 25, 28–34]. Additionally, X-VIVO has also been used for cultivating primary macrophages, providing valuable insights for the translation of results from different cell sources. Over a period of up to 12 weeks, the hemanoids constantly generated pre-macrophages that highly expressed macrophage-related surface markers such as CD14, CD163, CD11b and CD45, while lacking the pluripotency marker SSEA4 or granulocyte marker CD66b. The cell product demonstrated phagocytic capabilities by engulfing pHrodo™ BioParticles (Supplementary Figure S1A, B) and producing reactive oxygen species (ROS) after stimulation with phorbol 12-myristate-13-acetate (PMA) (Supplementary Figure S1C). Before applying the cells in different functional assays, we seeded the freshly harvested cells for three days terminal differentiation in either RPMI1640 medium supplemented with the animal serum FBS (RPMI + FBS), or continued cultivation in the commercial and chemically defined X-VIVO medium containing human serum albumin (hSA) by default, both supplemented with 50 ng/mL M-CSF (Fig. 1A). For some comparisons, a differentiation setting without FBS (RPMI-FBS) was also included.

After three days, the iPSC-Mac adhered to the surface of the cultivation plate and showed a typical roundish or elongated morphology. Remarkably, we observed a stronger elongation and planar distribution of the iPSC-Mac cultured under RPMI + FBS conditions during the cultivation of the cells, while the cells cultured with X-VIVO showed less elongation and adherence to the plate surface (Supplementary Figure S2). No morphological differences between the cells were observed in cytospin preparations (Fig. 1B). When comparing the cell count of all living cells three days post-seeding with the number of initially seeded cells, 5–25% cell loss occurred with no significant differences between X-VIVO and RPMI + FBS cultivation, although a higher standard deviation occurred for the RPMI + FBS differentiated iPSC-Mac. Under serum-lacking RPMI-FBS condition, however, we observed a significant loss of 68% ± 13% (iPSC line #1) and 55% ± 20% (iPSC line #2) cells compared to the initial number of seeded cells (*n* = 8) (Fig. 1C).

One major difference between the two different media was their glucose level (Fig. 1D). With approximately 650 mg/dL, X-VIVO medium contains roughly 3.25 times more glucose than RPMI medium (200 mg/dL). Nevertheless, the glucose consumption rates were comparable, as well as the lactate production over the 24 h indicating no tremendous metabolic differences (Fig. 1E).

iPSC-Mac were harvested after three days of terminal differentiation in each medium and analyzed for the expression of common macrophage surface markers. Most surface proteins, including CD163, CD14, CD11b, CD86, CD16, CD45, CD206 and HLA-DR showed very similar and comparable expression levels for both iPSC lines, independent of the medium condition (Fig. 1F, Supplementary Figure S3). Minor, but no significant differences were observed for the Fc gamma receptor Ia (CD64), which was slightly upregulated under X-VIVO condition. These observations align with others, demonstrating no impact of the cultivation medium on characteristic macrophage surface marker expression [19, 22–25]. Noteworthy, while total expression of the different surface markers was comparable between iPSC-Mac from both iPSC lines, remarkable differences were observed for CD14, which was roughly 2.8–3.9 times higher in iPSC line #2 compared to line #1, indicating some degree of donor heterogeneity in surface marker expression levels. iPSC-Mac from both terminal differentiation protocols generated high amounts of ROS after stimulation with PMA, but with significantly higher levels for X-VIVO differentiated iPSC-Mac in one iPSC line (Fig. 1G). This finding indicated a decreased responsiveness of iPSC-Mac to a pro-inflammatory stimulus when switched to RPMI + FBS medium.

### RPMI + FBS induces a more pro-inflammatory gene expression signature compared to X-VIVO cultivation

**Fig. 2:**
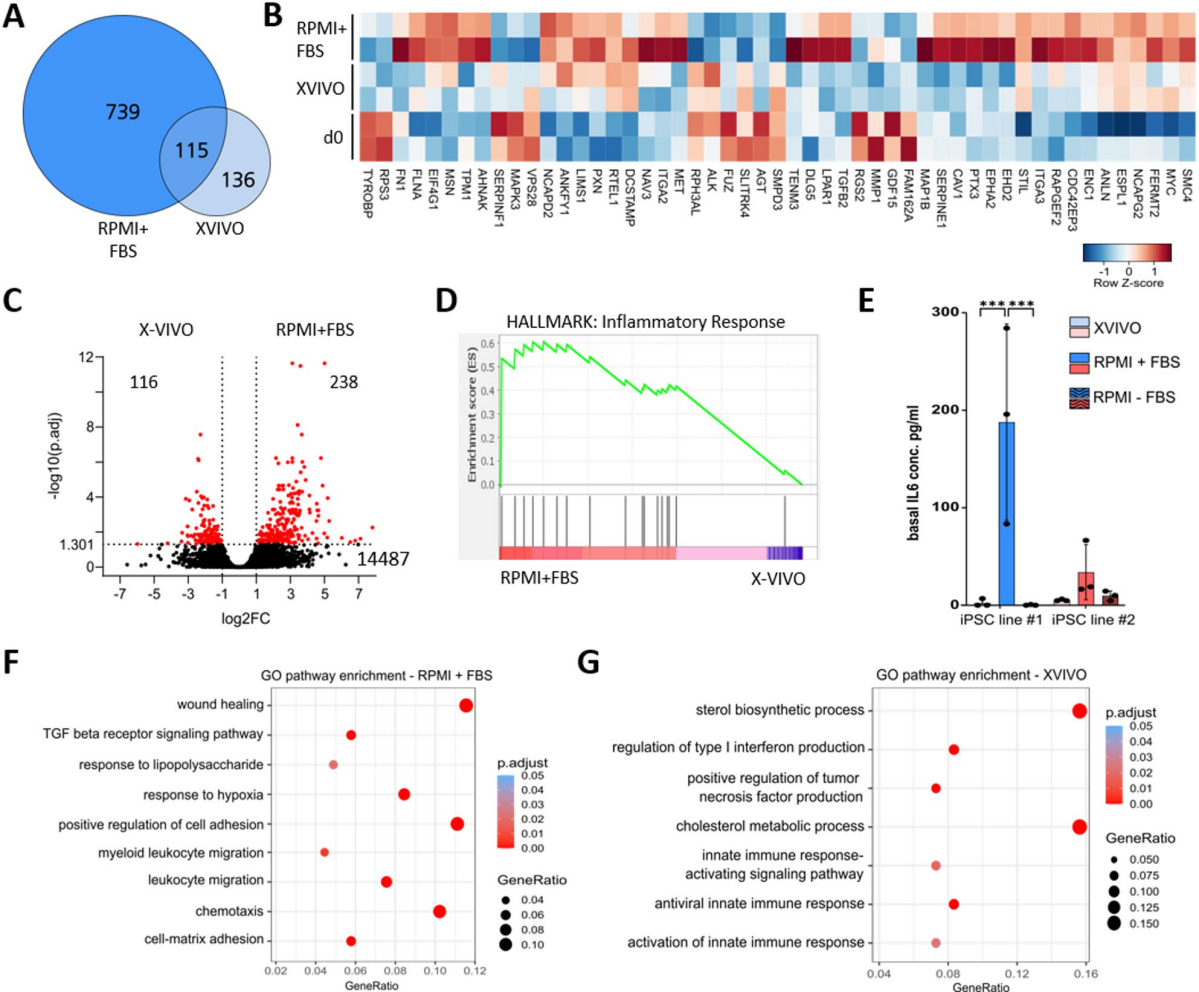
Transcriptional comparison of the terminal differentiation in X-VIVO or RPMI + FBS on iPSC line#2. **A** Venn diagram showing the number of deregulated genes between freshly isolated iPSC-Mac (d0) and terminally differentiated cells for three days either in RPMI + FBS or X-VIVO. **B** Hierarchical heat map clustering of chosen genes involved in cellular component organization (GO:0051130). **C** Volcano plot showing differentially expressed genes (DEG) between both media upon terminal differentiation (padj < 0.05, |log2FC| > 0.9), including the number of up-regulated genes for the respective medium, and non-significant DEGs. **D** Gene set enrichment analysis (GSEA) comparing 3 days terminal differentiation in RPMI + FBS vs. X-VIVO with regard to the hallmark gene set ‘Inflammatory response’ (NES 1.84, FDR q-value 0.173). **E** Basal expression of IL-6 measured in the supernatants post terminal differentiation in either X-VIVO, RPMI + FBS or RPMI-FBS. Graph shows mean ± SD. Statistical analysis was performed using two-way ANOVA with Tukey’s multiple comparison post-hoc test, *n* = 3. **F**, **G** Selection of significantly enriched biological processes following gene ontology (GO) enrichment analysis (padj < 0.05) ranked by the gene ratio after terminal differentiation in **(F)** RPMI + FBS or **(G)** X-VIVO

One of the key functions of macrophages is their ability to adapt their functional and metabolic behavior in response to different environmental cues. To assess this potential for the iPSC-Mac following terminal differentiation in our manufacturing process, we employed a transcriptome analysis on macrophages derived from iPSC-line #2. Due to technical challenges encountered with the other line, high-quality sequencing data could not be obtained. Harvesting freshly produced cells (d0) and following terminal differentiation resulted in an extensive change in gene expression with 854 differentially expressed genes (DEG, padj < 0.05, |log2FC| > 0.9) for RPMI + FBS condition and 251 DEG in XVIVO (Fig. 2A). Many of these genes were involved in antiviral innate immune response (GO:0140374) for X-VIVO condition, whereas profound changes in cell junction assembly (GO:0034329) or cell matrix adhesion (GO:0007160) were upregulated following RPMI + FBS cultivation (full list of GO analysis in Supplementary table S2). The extensive organization of cellular components (GO:0051130) was mostly upregulated under RPMI + FBS condition (Fig. 2B) highlighting more profound cellular changes for this terminal differentiation protocol. Comparing gene expression between both terminal differentiation strategies revealed 354 DEG with 238 genes being upregulated in RPMI + FBS and 116 genes in X-VIVO (Fig. 2C). By performing Gene Set Enrichment Analysis (GSEA) on the DEG, we identified an increased inflammatory response for RPMI + FBS terminally differentiated macrophages (Fig. 2D). To verify this finding, we measured the basal secretion of the pro-inflammatory cytokine IL-6 after three days of differentiation. As a control, the iPSC-Mac were also terminally differentiated in the absence of serum (RPMI-FBS). Only in the presence of the animal serum, higher IL-6 levels of 187.7 ± 82.1 pg/mL (iPSC line #1) were detected, whereas X-VIVO or RPMI-FBS only induced IL-6 production of 2.3 ± 3.3 pg/mL or 0.3 ± 0.4 pg/mL, respectively (Fig. 2E). This observation was true for iPSC-Mac from both iPSC lines, although differences in the total amount were observed between the two lines. Importantly, the considerable variability in IL-6 secretion observed in iPSC line #1 underscores the sensitivity of iPSC-Mac to experimental conditions and intrinsic cellular heterogeneity.

To further identify transcriptional differences, we performed enrichment analysis on the DEG and identified various enriched pathways between the two differentiation conditions. A striking set of genes included the ‘positive regulation of cell adhesion’ and ‘cell-matrix adhesion’ for FBS-grown iPSC-Mac (Fig. 2F). These gene sets were consistent with the increased cell adhesion and elongation observed during cultivation (Supplementary Figure S2). Another result was the enrichment of lipid-metabolic processes, including phospholipids, sterols and cholesterol after differentiation under X-VIVO conditions (Fig. 2G), which is consistent with previous literature investigating the impact of differentiation protocols [24, 27].

Further evaluation of the transcriptional differences revealed not only differences in metabolism and cell adhesion, but also important functional consequences on immune function. The previously described enhanced IL-6 secretion under FBS conditions was consistent with an upregulated ‘response to lipopolysaccharide’ gene set (Fig. 2F). Similar gene sets were implicated in immune activation and affected chemotaxis and migration of immune cells. However, X-VIVO medium apparently impacted the immune response as well, in a way that pathways like ‘innate immune response activating signaling pathway’ or ‘antiviral innate immune response’ were significantly upregulated (Fig. 2G). These pathways have in common that they include genes that respond to interferons, like *IFIT1*, *IFIT3*, *OAS1*, *RIG1*, *ISG15* (Supplementary table S2). However, the examined gene sets were derived from the direct comparison of X-VIVO- and RPMI + FBS-treated iPSC-Mac, meaning that the increased expression of genes involved in a respective gene set does not necessarily mean an activation through stimulation, but could also indicate the absence or a decreased activity within the other media treatment. In this line, we assumed that the FBS-containing medium pre-activated the iPSC-Mac, leading to a prior immune activation. Following this assumption, we hypothesized that the preceding activation could lead to desensitization to new external stimulation.

### Differentiation in the presence of animal serum desensitizes iPSC-Mac upon external stimulation

**Fig. 3:**
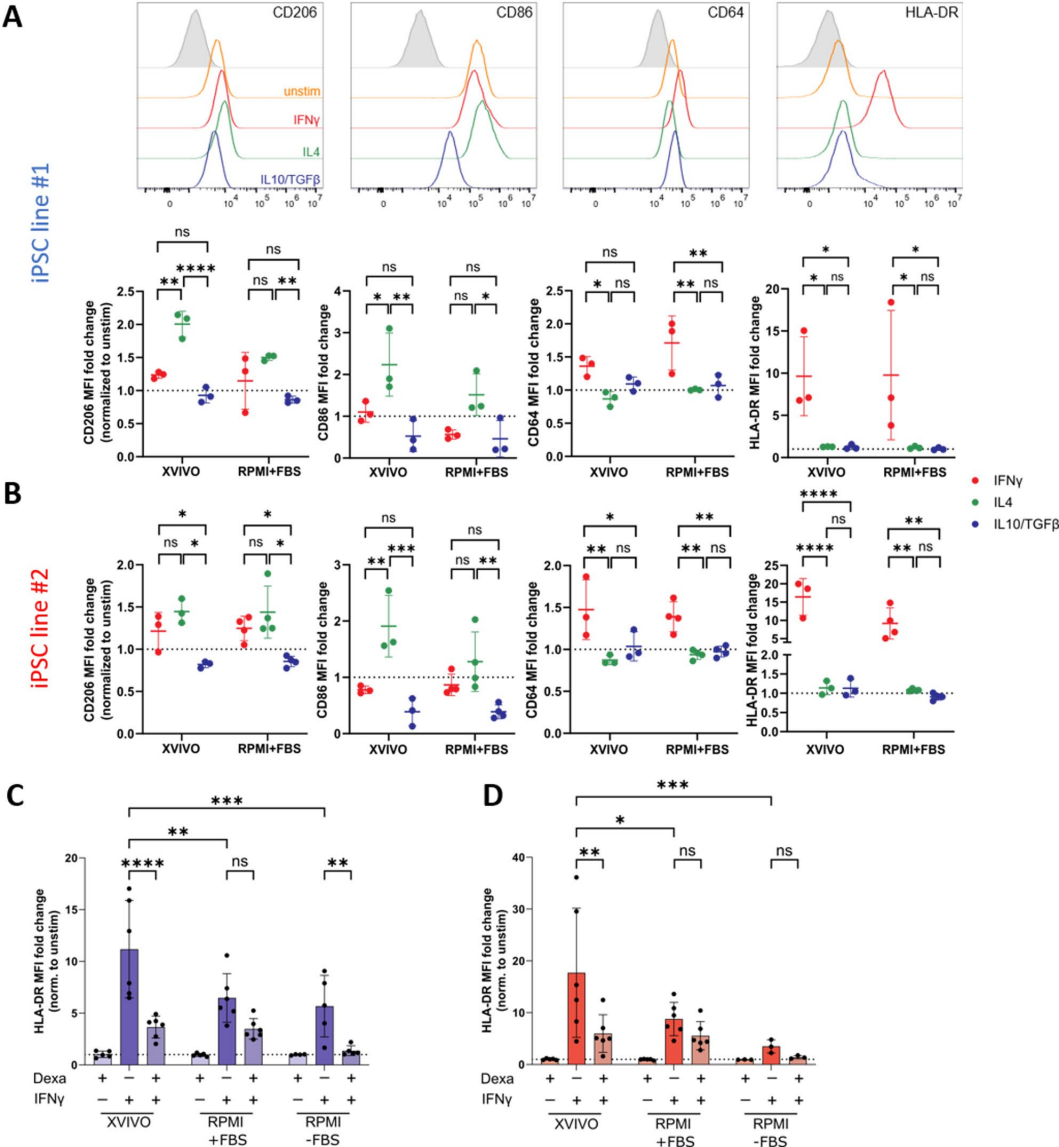
iPSC-Mac respond differently towards external stimulation depending on the cultivation condition. iPSC-Mac were terminally differentiated for three days either in X-VIVO or RPMI + FBS medium. **A**, **B** Cells from both iPSC **(A)** line #1 and **(B)** line #2 were treated with either 25 ng/mL IFNγ, 10 ng/mL IL-4 or 10 ng/mL IL-10/TGFβ. Depicted are representative histograms and dot plots showing individual median fluorescence intensities (MFI) after normalization to the respective unstimulated (unstim) control for the macrophage activation markers CD206, CD86, CD64 and HLA-DR. Graphs show mean ± SD. Statistical analysis was performed using two-way ANOVA with Tukey’s multiple comparison post-hoc test, *n* = 3. **C**, **D** iPSC-Mac from both iPSC **(C)** line #1 and **(D)** line #2 were differentiated in the two aforementioned ways or for three days in RPMI medium without FBS (RPMI-FBS). Stimulation was performed with 5 ng/mL IFNγ with or without simultaneous (co-) stimulation with 1 µg/mL dexamethasone (Dexa) for 24 h. Expression of HLA-DR was determined by flow cytometry and depicted as histograms after normalizing the MFI to the unstimulated control condition, *n* = 3–6

While other research groups have already investigated the influence of different media on the differentiation and metabolic activity of iPSC-derived macrophages [19, 24, 27], this study specifically focuses on the impact of two differentiation platforms on the immunological activity of macrophages and their suitability in different functional assays. Following our hypothesis that medium components interact and pre-stimulate the iPSC-Mac, we were interested in the cell’s behavior upon additional stimulatory factors. Thus, we treated the terminally differentiated iPSC-Mac with different cytokines to induce distinct polarization states and examined the expression changes in common surface proteins. In line with a previous description on polarization [40], we treated iPSC-Mac either with 25 ng/mL IFNγ to induce a pro-inflammatory M1(IFNγ) phenotype, 10 ng/mL IL-4 for an anti-inflammatory M2(IL-4) phenotype, or 10 ng/mL IL-10 together with 10 ng/mL TGFβ for a more quiescent, anti-inflammatory M2(IL-10/TGFβ) phenotype. Following 24 h of stimulation, the common polarization markers CD206, CD86, CD64 and HLA-DR were measured via flow cytometry. To compare the strength of the phenotypic change between the different groups, the median fluorescence intensity (MFI) was normalized to the unstimulated control (Fig. 3A, B). iPSC-Mac that were cultivated in X-VIVO demonstrated less pre-activation and therefore responded by more pronounced changes in their surface marker expression to either the pro- or anti-inflammatory polarization state. This was marked by a 2- and 1.45-fold upregulation of CD206 after the anti-inflammatory IL-4 treatment and was even more profound for CD86 expression (2.23- and 1.51-fold for iPSC lines #1 and #2). While often considered as a pro-inflammatory marker, IL-4 stimulation upregulates CD86 in iPSC-derived macrophages, as reported in previous studies [19, 40]. We confirmed this expression pattern, however, a significant difference between IL-4 and IFNγ-treatment was only observed in the X-VIVO setting. Stimulation in the serum-containing RPMI + FBS medium resulted in reduced phenotypic changes of CD206 (1.5- and 1.44-fold) and CD86 (1.51- and 1.28-fold) upon IL-4 treatment, supporting the pre-activation theory. The upregulation of CD206 observed in our iPSC-Mac closely mirrors the levels reported by others [22, 24], although some studies have shown substantially higher CD206 induction under similar differentiation conditions, underlining that iPSC line-specific factors influence the marker expression magnitude [41]. For the two pro-inflammatory surface markers CD64 and HLA-DR, no striking differences have been observed upon IFNγ stimulation between the two media tested. Also, IL-10/TGFβ treatment was sufficient to downregulate the activation markers CD206 and CD86, independent of the differentiation protocol (Fig. 3A, B).

Following this observation, we concluded that serum selection plays an important role when examining drug responses on iPSC-Mac and hence needs to be carefully considered in the design of the downstream study especially for immunomodulatory drugs. Ensuring a proper response of iPSC-Mac towards compounds is crucial for any model system testing the efficacy of immunomodulatory drugs. In a next approach, we therefore investigated the impact of animal serum on the reactivity of a pre-activated iPSC-Mac to an anti-inflammatory treatment. Therefore, cells were stimulated with 5 ng/mL IFNγ to induce an upregulation of HLA-DR. As an anti-inflammatory treatment, the cells were additionally stimulated with 1 µg/mL dexamethasone, which interfered with the upregulation of HLA-DR [22]. Regardless of the iPSC line used, iPSC-Mac derived from X-VIVO cultures and treated with IFNγ showed a much stronger response by upregulating HLA-DR compared to RPMI + FBS-cultivated cells. Notably, while dexamethasone treatment could reverse this phenotype, again X-VIVO-grown cells showed a much more pronounced phenotype compared to cells from RPMI + FBS cultivation (3.06-fold and 2.96-fold decrease versus 1.86-fold and 1.57-fold decrease for iPSC lines #1 and #2, respectively), highlighting the sensitivity of these cells and the presumably interfering effect of FBS (Fig. 3C, D). Surprisingly, a significant preventive effect of dexamethasone on HLA-DR upregulation (4.12-fold) was also observed in macrophages from iPSC line #1 under serum-free RPMI conditions (Fig. 3C), although at much lower total expression levels compared to X-VIVO. This highlights the impact of FBS on iPSC-Mac by repressing response to co-stimulatory signals.

### The absence of serum makes iPSC-Mac more responsive to stimulation

**Fig. 4:**
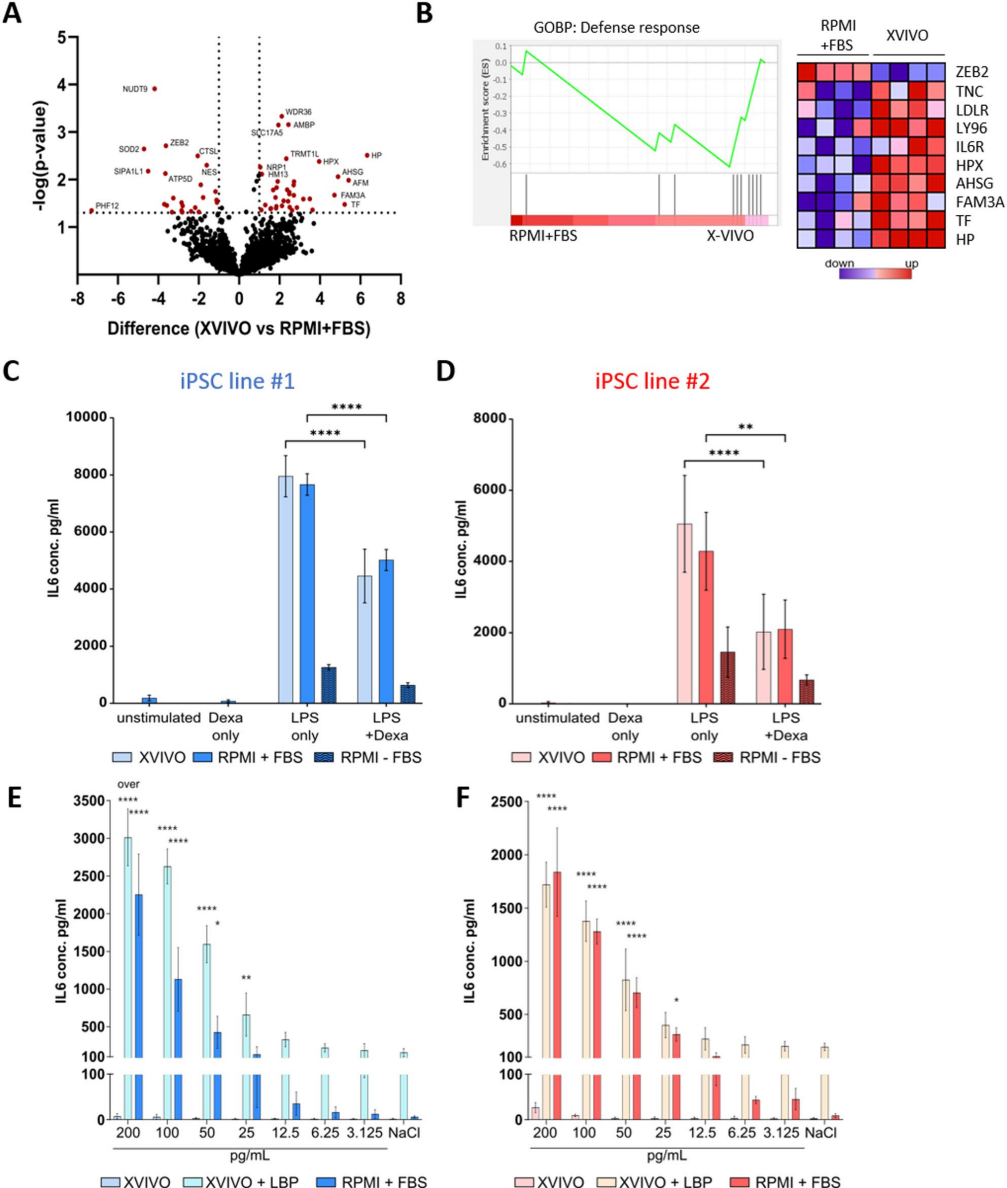
The cultivation condition influences the cell surface receptor expression on iPSC-Mac and subsequently the sensitivity towards LPS stimulation. iPSC-Mac were terminally differentiated for 3 days in either X-VIVO or RPMI + FBS. **A**, **B** Protein lysates from two biological iPSC-Mac replicates from both iPSC lines were isolated and enriched for glycosylated proteins and measured by mass spectrometry. **A** Vulcano plot showing differentially expressed proteins that were confidently identified in both replicates. Proteins were considered as significantly altered (red dot) if the p-value was below 0.05. **B** GSEA was performed on all significantly deregulated proteins between RPMI + FBS and X-VIVO differentiation with regard to the gene ontology biological processes gene set ‘Defense response’. The left plots the enrichment, while the right shows a heatmap with z-score normalized expression values (blue – low expression, red – high expression) (NES − 1.36, p-value 0.166, FDR q-value 1.0). **C**, **D** Responsiveness of iPSC **(C)** line #1 and **(D)** line #2 to immunomodulatory drugs upon differentiation measured by released IL-6 levels after 4 h LPS stimulation. iPSC-Mac were treated with 100 ng/mL lipopolysaccharide (LPS) with or without additional stimulation with 1 µg/mL dexamethasone (Dexa). As an additional control, iPSC-Mac were differentiated and treated in the absence of serum (RPMI-FBS). Graphs show mean ± SD. Statistical analysis was performed using two-way ANOVA with Tukey’s multiple comparison post-hoc test, *n* = 3. **E**, **F** Dose-escalation treatment of LPS to X-VIVO, X-VIVO + LPS-binding protein (LBP) or RPMI + FBS treated iPSC-Mac for the iPSC **(E)** line #1 and **(F)** line #2. Histograms show IL-6 levels after 24 h. Graphs show mean ± SD. ‘over’ means the measured ODs were partly out of the range of the standard. Statistical analysis was performed using two-way ANOVA with Dunnett’s multiple comparison post-hoc test, *n* = 3 (iPSC line #2) or 4 (iPSC line #1)

From the previous observation, we concluded that stimulant triggers in FBS can impair the iPSC-Mac receptivity to new stimulatory signals. We hypothesized that this pre-stimulation may alter the abundance of cell surface receptors that facilitate the recognition of novel stimulants, impacting their responsiveness. To evaluate the presence of relevant cell surface proteins, we conducted cell surface proteome (surfaceome) analysis after three days of terminal differentiation either in X-VIVO or RPMI + FBS for both iPSC lines in biological duplicates. To detect relevant proteins expressed on the cell surface, we enriched glycosylated proteins from cell lysates and performed mass spectrometry. Using this method, we identified in total 2693 proteins, of which 766 proteins were annotated glycoproteins by UniprotKB Keywords (PTM). From all identified proteins, 1028 different proteins allocated to the plasma membrane, followed by 837 proteins that belonged to the Golgi apparatus or the endoplasmic reticulum, and 814 proteins being part of the nucleus membrane (Supplementary Figure S4A). While we found large differences in expression between the two different iPSC lines (Supplementary Figure S4B), we identified common proteins that were differently expressed between both medium conditions (Fig. 4A). We then performed GSEA and identified a reduced but non-significant expression of immune-relevant proteins, involved in defense response (NES − 1.36, nominal p-value 0.166, FDR q-value 1.0; Fig. 4B). These proteins included various receptor and scavenger proteins, like LDLR, LY96, IL6R, TF, HP and HPX that were decreased in RPMI + FBS-differentiated iPSC-Mac.

Due to the increased receptor expression under X-VIVO conditions, including LY96, which is involved in LPS detection [42], we hypothesized an increased pro-inflammatory response in X-VIVO differentiated iPSC-Mac upon stimulation with 100 ng/mL LPS. Similarly to the previous experiments with IFNγ, one condition included the simultaneous treatment with 1 µg/mL dexamethasone, addressing immunomodulatory screening options. Irrespective of the differentiation protocol, iPSC-Mac responded by secreting large amounts of IL-6 upon LPS exposure. When additionally applying dexamethasone, the amount of secreted IL-6 was significantly reduced for both conditions, without relevant differences (Fig. 4C, D). Noteworthy, iPSC-Mac that were differentiated in serum-free RPMI secreted 2.4–6.3 times less IL-6 after LPS stimulation.

To further address the sensitivity of iPSC-Mac to stimulatory signals, we applied a dose-escalation treatment of LPS to X-VIVO or RPMI + FBS differentiated iPSC-Mac. Previous research work demonstrated that iPSC-Mac exhibit increased sensitivity towards endotoxins in the monocyte-activation test compared to primary human macrophages [16]. While we could not observe any difference in the pro-inflammatory response at 100 ng/mL LPS between both medium conditions, we investigated the secretion of IL-6 in a dose-escalation response using LPS doses in the range of 200 pg/mL to 3.125 pg/mL (thus 500–32,000-fold less). Of note, already at the dose of 200 pg/mL we were unable to observe a proper IL-6 secretion by X-VIVO-grown iPSC-Mac (Fig. 4E, F), suggesting a lack of co-stimulatory signals required for an appropriate response to LPS. In fact, addition of accessory proteins such as LPS-binding protein (LBP) mediated a proper immune response presumably facilitated by the CD14-TLR4-LY96 receptor complex (Fig. 4E, F) [42]. Irrespective of the iPSC line used and the differentiation conditions, human iPSC-Mac showed a dose-dependent response to LPS with decreasing amounts of IL-6 secreted into the supernatant. Albeit the background secretion in the presence of LBP, we observed a higher responsiveness between 200 pg/mL and 50 pg/mL for iPSC line #1. Concentrations below 25 pg/mL resulted in a detectable but noticeable reduction in the IL-6 response, which had previously been described for primary MDMs [16, 43]. Discrimination at lower concentrations of LPS was more sophisticated for RPMI + FBS conditions, still highlighting the sensitivity of iPSC-Mac in general (Supplementary Figure S5).

Overall, general functionality of iPSC-Mac is not affected by both differentiation protocols. However, when using the cells to precisely measure and modulate immune response X-VIVO medium enables iPSC-Mac to respond more extensively to different pro- and anti-inflammatory stimuli.

## Discussion

Various studies have shown the limitations of FBS in in vitro studies. In the context of iPSC-derived immune cells, in which every single step of differentiation - from the stem cell to the hematopoietic organoid - is very precisely defined, it seems all the more surprising when FBS is again used for the final differentiation step. In 2012, Rey-Giraud et al. already compared traditionally grown primary monocyte-derived macrophages (MDM) in RPMI + FBS to X-VIVO™ 10 medium [44]. They observed increased TNFα release in pro-inflammatory M1 macrophages using X-VIVO™ 10 medium compared to FBS-grown cells and a greater phagocytic property of M2 macrophages. Recently, few groups have compared different alternatives to less-defined protocols for iPSC-Mac [19, 24, 27]. The present study contributes to these works by highlighting inherent limitations of FBS for cell differentiation, which is still widely used in many studies on macrophages. Our results show that the limitations go beyond known problems for FBS like its batch-to-batch variability [45]. Terminal differentiation in RPMI + FBS leads to significant and fundamental changes in iPSC-Mac behavior. This process profoundly alters gene transcription, surface marker expression and physiological responses to external triggers and immunomodulatory influences compared to the continuous usage of X-VIVO medium, which can have tremendous impact when using iPSC-Mac for pharmacological examinations on immunomodulatory drugs as well as for toxicological applications. These effects were consistent across two different iPSC lines, although we observed variability in overall expression levels and responsiveness between donors. While these differences reflect donor heterogeneity- albeit more controlled than with primary cells - the reproducibility across different harvests highlights the robustness of the protocol.

In the past, several groups have compared bovine and human serum and their effects on different cell types. The work of Heger et al. showed improved spheroid formation and cell invasion of SiHa cells (cervical squamous cell carcinoma) for human serum [46], while others described varying levels of IL-8 or IL-6 secretion depending on FBS batches and concentration [35, 36]. These effects may influence data interpretation, leading to an over- or underestimation of a cell’s response. On top, Tang et al. have shown that animal serum compartments can impact physiological reactions already before cells are getting involved due to its interaction with compounds that are being tested [47]. When studying antimicrobial peptides, their group observed the binding and subsequent inhibition of various compounds to a complex of fetal bovine serum albumin and α1-antitrypsin. However, this complex formation has not been described for human serum or even adult bovine serum. The high prevalence of fetal serum may therefore lead to the false-negative exclusion of potential antimicrobial drug candidates [47]. Importantly, since X-VIVO per se contains hSA, we did not include a RPMI + hSA condition but focused exclusively on the two different established terminal differentiation protocols.

Another known effect of FBS is its contribution to cell attachment by providing proteins such as fibronectin or vitronectin [48, 49]. Consistent with this knowledge, we observed reduced cell attachment in X-VIVO medium containing only hSA, compared to iPSC-Mac cultivated with FBS. Although the cells robustly responded to external stimulants, the impaired cell attachment could alter cell signaling and should therefore be taken into account when considering other applications for iPSC-Mac, such as toxicological evaluation of biomaterials [50] or therapeutic evaluation of scaffolds for wound healing and tissue engineering.

Consistent with previous studies on iPSC-Mac differentiation using X-VIVO medium, our findings demonstrate increased type I interferon production and an enhanced cholesterol metabolism in differentiated iPSC-Mac compared to RPMI + FBS medium. Similar observations have been reported for X-VIVO medium and a chemically defined open-source medium (OXM) during differentiation when compared to HPLM medium [27]. Notably, both metabolic programs are associated with improved cellular sensitivity and responsiveness. Type I interferons are known to improve inflammatory functions by preparing the chromatin in such a way that the silencing of target genes regulated by the transcription factor nuclear factor kappa-light-chain-enhancer of activated B-cells (NF-κB) is prevented [51]. This effect enables strong transcriptional responses even to weak inflammatory signals. Concurrently, elevated cholesterol levels in macrophage plasma membranes have been shown to augment TLR4 signaling, while increased free cholesterol levels in endosomes enhances TLR3 signaling. Thus, cholesterol accumulation can potentiate responses to external TLR ligands [52].

As demonstrated within this work, one limitation of a serum-free, chemically defined medium such as X-VIVO™ 15 is the reduced sensitivity of iPSC-Mac to low concentrations of LPS. While high concentrations effectively induced cellular signaling and promoted IL-6 secretion, lower concentrations that were more physiologically relevant failed to elicit an adequate immune response. Both animal and human serum contain carrier molecules that facilitate LPS binding to the CD14-TLR4-LY96 receptor complex [53]. In this study, we demonstrated that the incorporation of LBP restores LPS detection, subsequently leading to an even higher IL-6 secretion, compared to RPMI + FBS-cultivated iPSC-Mac, although with higher background levels. The addition of LBP to X-VIVO resulted in IL-6 production comparable to that seen in primary MDMs in other studies [54]. LBP serves thereby not only as a binding partner for LPS, but can also enhance intracellular trafficking following internalization, highlighting its critical role in facilitating an appropriate immune response [55]. Nevertheless, the present work only investigated one batch of FBS and its impact on one TLR4 agonist. It did not explore other TLR signaling inducers that may not require carrier molecules.

## Conclusion

We conclude that terminal differentiation in the presence of FBS desensitizes iPSC-Mac due to the high abundance of bioactive molecules such as growth factors, hormones and cytokines. The underlying basal activation leads to a reduced cellular response to additional stimulants that trigger both pro- and anti-inflammatory activation modes. This fact has explicit consequences for the use of macrophages in pharmacological studies. iPSC-Mac differentiated in X-VIVO medium exhibit improved responsiveness to novel stimuli and do not show this limitation due to the absence of undefined bioactive molecules as present in FBS. However, the absence of these co-stimulatory factors requires careful evaluation and validation, particularly when used for endotoxin or non-endotoxin testing. While X-VIVO 15™ is a chemically defined medium, its proprietary nature increases costs and complicates the identification of necessary additives for toxicity assays. Recently published open-source, serum-free media may therefore represent a highly effective alternative for such applications.

## Supplementary Information

Below is the link to the electronic supplementary material.

Supplementary Material 1.

Supplementary Material 2.

Supplementary Material 3.

## Data Availability

The dataset supporting the conclusions of this article are included within the article and its additional files. The RNA sequencing data presented in the study is deposited in the GEO repository with the accession number GSE311159. Raw mass spectrometry data have been deposited to the ProteomeXchange Consortium via the PRIDE partner repository (http://www.ebi.ac.uk/pride) with the dataset identifier PXD070961.

## Abbreviations

ABC: Ammonium hydrogencarbonate
ANOVA: Analysis of variance
Surfaceome: Cell surface proteome
DFG: Deutsche Forschungsgemeinschaft
Dexa: Dexamethasone
DEG: Differentially expressed genes
DHR: Dihydrorhodamine 123
DPBS: Dulbecco’s Balanced Salt Solution
DMEM: Dulbeccos Modified Eagle Medium
DMEM/F-12: Dulbecco’s Modified Eagle Medium/Nutrient Mixture F-12
EB: Embryoid body
E6: Essential 6
E8: Essential 8
EDTA: Ethylenediaminetetraacetic acid
ERC: European Research Council
CD64: Fc gamma receptor Ia
FBS: Fetal bovine serum
GO: Gene ontology
GSEA: Gene set enrichment analysis
HBSS: Hanks’ Balanced Salt Solution
HCD: Higher energy collisional dissociation
hbFGF: Human basic fibroblast growth factor
hBMP4: Human bone morphogenetic protein 4
IFNy: Human interferon-gamma
hM-CSF: Human macrophage colony stimulating factor
HPLM: Human platelet lysate medium
hSCF: Human stem cell factor
hTGFb1: Human transforming growth factor beta 1
iPSC: Induced pluripotent stem cells
IL-10: Interleukin 10
IL-3: Interleukin 3
IL-4: Interleukin 4
IL-6: Interleukin 6
iPSC-Mac: iPSC-derived macrophages
KO: knockout
LFQ: Label-free quantification
LPS: Lipopolysaccharide
LBP: Lipopolysaccharide-binding protein
MFI: Median fluorescence intensity
MAT: Monocyte Activation Test
MDM: Monocyte-derived macrophages
MCFC: Myeloid cell forming complexes
NF-κB: Nuclear factor kappa-light-chain-enhancer of activated B-cells
PPR: Pattern recognition receptors
PMA: Phorbol myristate acetate
ROS: Reactive oxygen species
RI: ROCK-Inhibitor
RPMI: Roswell Park Memorial Institute Medium
NaCl: Sodium chloride
TRM: Tissue-resident macrophages
TLR: Toll-like receptors
TNFα: Tumor necrosis factor alpha
hVEGF: Vascular endothelial growth factor
WHO-LPS: WHO 3rd international endotoxin standard
X-VIVO: X-VIVO™ 15 medium
