# Supplementary material for: Defined culture conditions improve functional properties of mature iPSC-derived macrophages for therapeutic screening
Source: Stem Cell Res Ther. 2026 Apr 11;17:134. doi: 10.1186/s13287-026-05009-1 (PMC13078047; doi:10.1186/s13287-026-05009-1)
Supplement: Supplementary file 1 — Supplementary Material 1. [file 13287_2026_5009_MOESM1_ESM.docx]

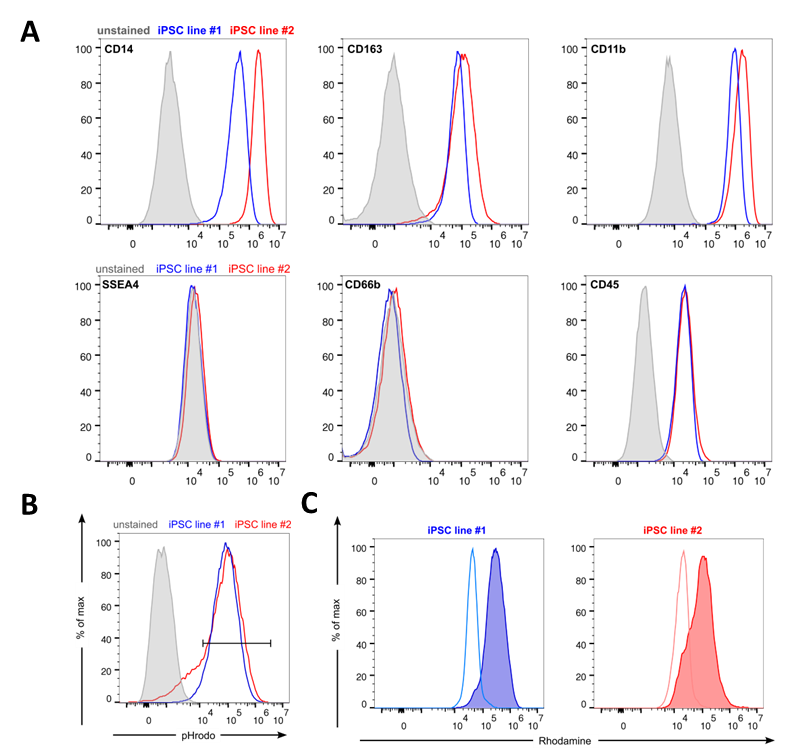


**Supplementary Figure S1: iPSC-Mac characterization after harvesting.** **(A)** Phenotypic characterization of iPSC-Mac from two different iPSC lines. Representative histograms show expression of the typical macrophage surface markers CD14, CD163, CD11b, CD45 and the absence of SSEA4 and CD66b for iPSC line #1 (blue), iPSC line #2 (red) and unstained (grey). **(B)** Phagocytosis of pHrodo™ Red E. coli BioParticles™ after 2h cultivation at 37 °C. **(C)** Reactive oxygen species (ROS) production from iPSC-Mac derived from two iPSC lines. Depicted are samples stained with Dihydrorhodamine 123 (line) and samples with the additional stimulation with phorbol myristate acetate (filled).


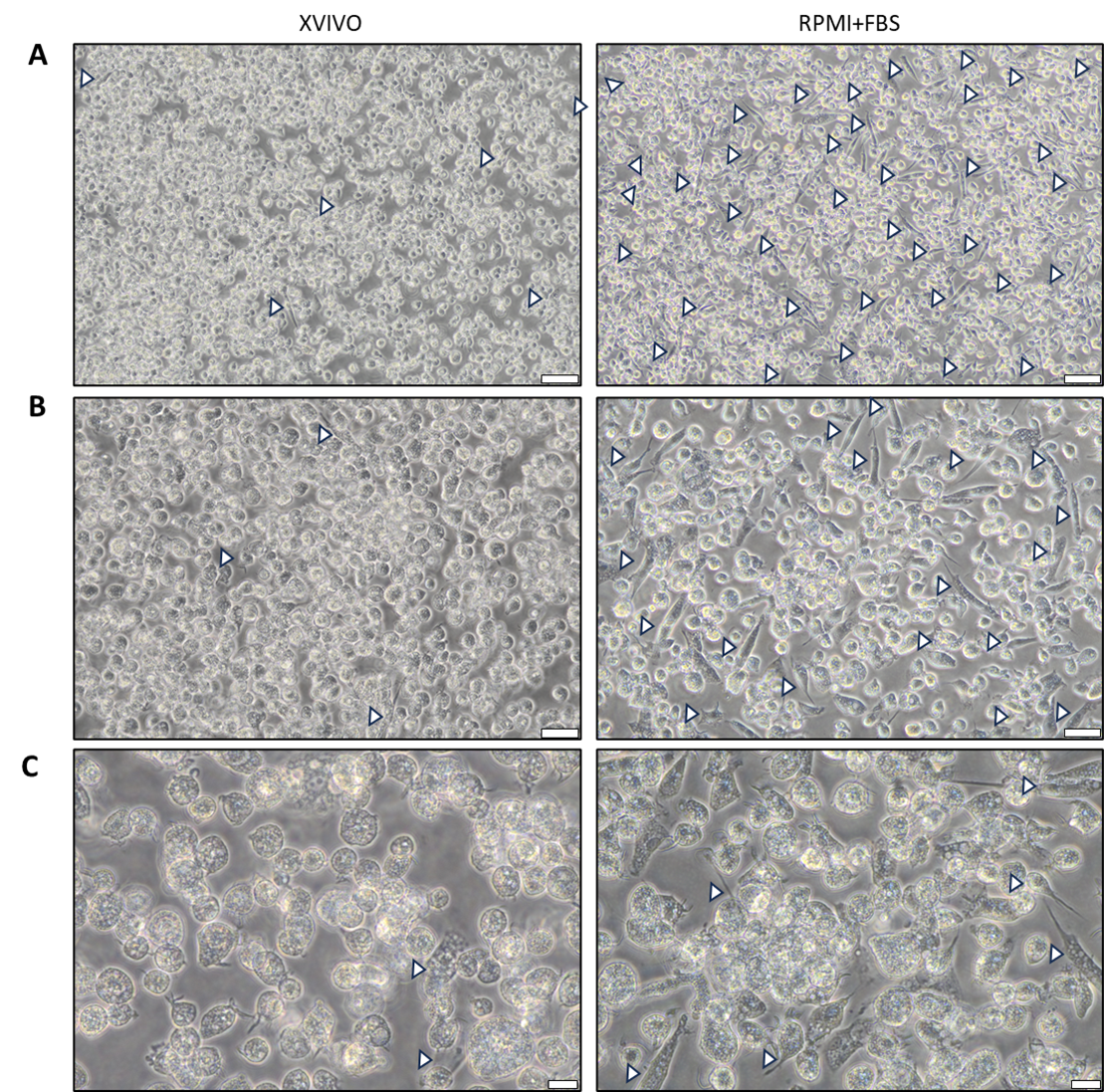


**Supplementary Figure S2: Morphological analysis post terminal differentiation.** Representative microscopic pictures at day three of terminal differentiation in X-VIVO medium or RPMI+FBS medium. Arrows indicate elongated cells attached to the culture surface. Magnification **(A)** 10x, scale bar – 100 µm, **(B)** 20x, scale bar – 50 µm and **(B)** 40x, scale bar – 20 µm.


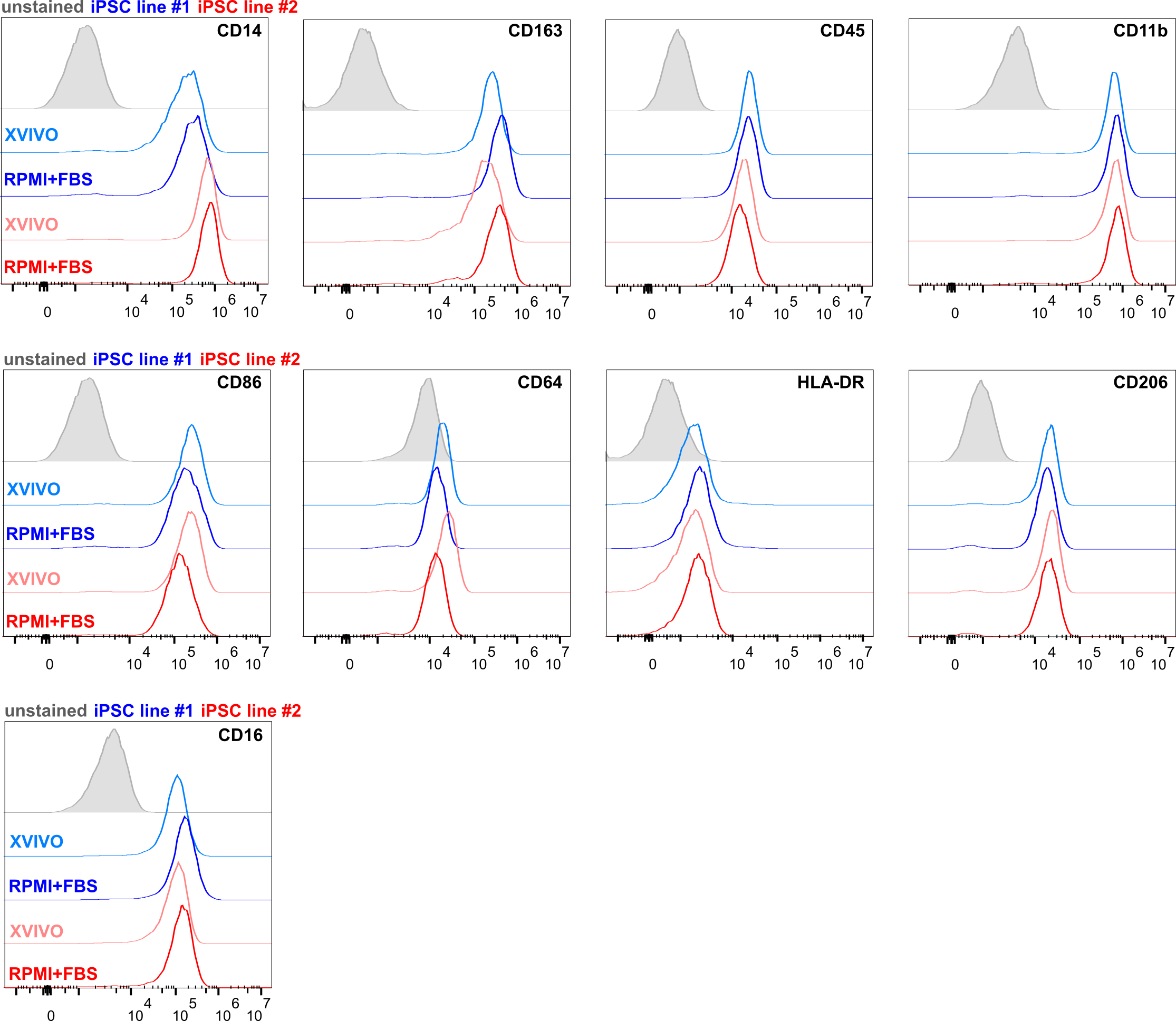


**Supplementary Figure S3: iPSC-Mac characterization after terminal differentiation.** Phenotypic characterization of iPSC-Mac from two different iPSC lines post three day terminal differentiation in X-VIVO medium (bright color) or RPMI+FBS medium (dark color). Representative histograms show expression of the typical macrophage surface markers CD14, CD163, CD11b, CD45, CD86, CD64, HLA-DR, CD206 and CD16 for iPSC line #1 (bluish), iPSC line #2 (reddish) and unstained (grey).

**
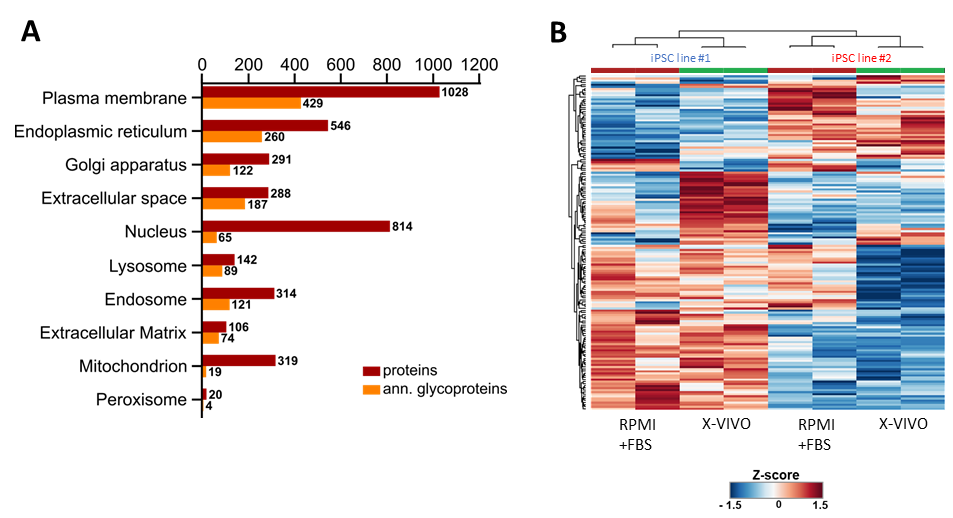
**

**Supplementary Figure S4: Overview of proteome data.** **(A)** Overview of the number of detected N-linked glycopeptides via mass spectrometry with proteins identified in both replicates of each condition. Assignment of all identified proteins to subcellular localization and classification as a glycoprotein was based on annotated glycoproteins by UniprotKB Keywords. **(B)** Unsupervised heatmap with hierarchical clustering of normalized, significantly deregulated proteins (ANOVA, p-value < 0.05) for two replicates of two iPSC lines after three days of terminal differentiation in X-VIVO (green) or RPMI+FBS (red) medium.


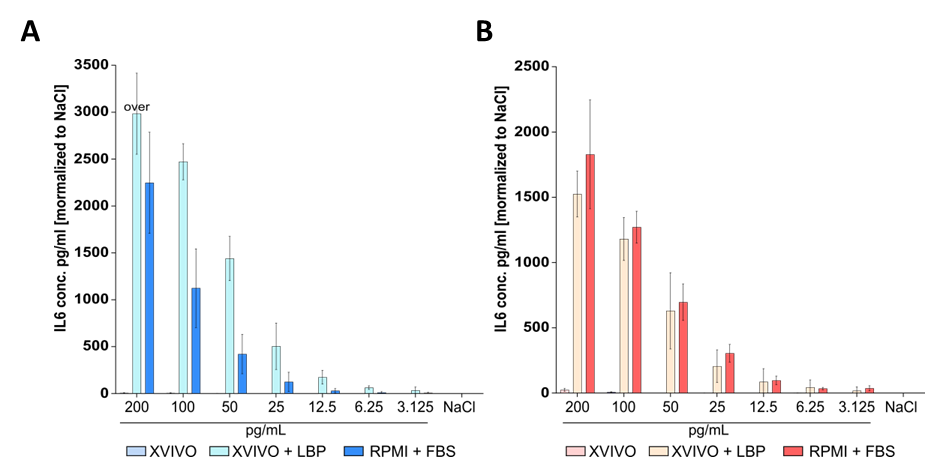


**Supplementary Figure S5: Normalized dose-escalation treatment of LPS.** Re-scaled picture from Figure 4E+F. iPSC-Mac from iPSC **(A)** line #1 and **(B)** line #2 were terminally differentiated for 3 days in either X-VIVO or RPMI+FBS and stimulated with decreasing concentrations of LPS in X-VIVO, X-VIVO + LPS-binding protein (LBP) or RPMI+FBS medium. Histograms show IL-6 levels after subtraction of background levels (NaCl) after 24h (n=3). Graphs show mean ± SD. ‘over’ means the measured ODs were partly out of the range of the standard.
